# The novel transcriptional activator Bhr1 combining NTPase and Zn(II)2Cys6 DNA-binding domains controls (hemi-)cellulase response to mannose-rich substrates in the white-rot fungus *Dichomitus squalens*

**DOI:** 10.1128/spectrum.00736-26

**Published:** 2026-07-24

**Authors:** Victor M. Gonzalez Ramos, Anna Lipzen, Hope Hundley, Vivian Ng, Igor V. Grigoriev, Miia R. Mäkelä

**Affiliations:** 1 Department of Bioproducts and Biosystems, Aalto University, Espoo, Finland; 2 Department of Microbiology, University of Helsinki 3835, Helsinki, Finland; 3 US Department of Energy Joint Genome Institute, Lawrence Berkeley National Laboratory 1666, Berkeley, California, USA; 4 Department of Plant and Microbial Biology, University of California Berkeley 118549, Berkeley, California, USA; USDA-ARS-NPRL, Dawson, Georgia, USA

**Keywords:** basidiomycete, transcriptional regulator, (hemi-)cellulases, CRISPR/Cas9, transcriptomics, phylogenetic analyses

## Abstract

The regulatory landscape responsible for lignocellulose degradation in white-rot basidiomycete fungi remains largely unexplored. In this study, we characterize a novel transcriptional activator, Bhr1, in the white-rot fungus *Dichomitus squalens*. Bhr1 exhibits an unusual domain architecture that combines a septin-like P-loop NTPase fold with Zn(II)2Cys6 DNA-binding domains and plays a critical role in activating (hemi-)cellulase enzyme production when *D. squalens* is exposed to mannose-rich substrates. Using CRISPR/Cas9-mediated gene editing, we generated a *bhr1* disruption mutant that displayed distinct phenotypes and enzyme activity profiles on mannose and guar gum compared to the wild type. RNA sequencing data indicate that Bhr1 induces specific (hemi-)cellulase-encoding genes without altering the expression of genes encoding sugar transporters or sugar metabolic enzymes. Phylogenetic analyses show that Bhr1 is basidiomycete specific and largely restricted to saprotrophic and plant-associated Agaricomycetes fungi. Based on the domain architecture of Bhr1 and the effects of its disruption in *D. squalens*, our findings reveal a lineage-specific regulatory innovation in basidiomycetes that is distinct from those described in ascomycetes. Elucidating the function and evolutionary conservation of Bhr1 advances our understanding of lignocellulose degradation at the molecular level in basidiomycete fungi and may inform studies of their ecological adaptation and the development of biotechnological applications.

**IMPORTANCE:**

Understanding the transcriptional regulatory mechanisms in white-rot fungi, such as *Dichomitus squalens*, is crucial for advancing our knowledge of lignocellulose degradation. This study identifies *D. squalens* Bhr1 as a key regulator of (hemi-)cellulase production on mannose-rich substrates and further distinguishes basidiomycete transcription factors involved in plant biomass degradation from their ascomycete counterparts. Our findings highlight the significance of lineage-specific regulators in facilitating adaptive enzyme production for efficient biomass utilization, which is critical to carbon cycling in terrestrial ecosystems. This work establishes a foundation for exploring novel regulatory strategies among wood-degrading fungi, potentially enabling targeted strain engineering in biotechnological applications.

## INTRODUCTION

Fungi are among the most important decomposers in terrestrial ecosystems, driving plant biomass turnover and sustaining the global carbon cycle (1). By tailoring their production of carbohydrate-active enzymes (CAZymes) (2), they depolymerize the major components of the plant cell wall, i.e., cellulose, hemicelluloses, pectin, and lignin, into simple sugars and aromatic monomers that can be assimilated or further metabolized (3, 4). CAZyme production is tightly regulated at the transcriptional level, allowing fungi to rapidly adapt to the composition of available substrates (5).

In filamentous ascomycetes, several genes responsible for transcriptional regulation of plant biomass degradation have been characterized (6). Zn(II)2Cys6-type transcription factors (TFs) are a major group of regulators that modulate the expression of CAZyme-encoding genes in response to lignocellulose-derived mono- and disaccharides and their metabolic products (5, 7). For example, XlnR, initially characterized in *Aspergillus niger*, activates cellulase- and xylanase-encoding genes together with xylose metabolic genes in response to xylose and its downstream metabolites (8). While XlnR orthologs are widespread in filamentous ascomycete fungi, their function varies from species to species (9). AraR, an XlnR homolog restricted to selected Eurotiales species, controls arabinose utilization pathways (10). In *Neurospora crassa*, CLR-2 is a critical activator of cellulase- and selected sugar transporter-encoding genes in response to cellulose (11). However, the CLR-2 ortholog is not essential for cellulase induction in *Trichoderma reesei*, where this role is fulfilled by other regulators, including the activator Ace3 (12). In addition to these activators, expression of CAZyme-encoding genes is constrained by carbon catabolite repression, mediated by the conserved C2H2 zinc-finger repressor CreA/Cre1, which prevents their expression in the presence of preferred carbon sources such as D-glucose (13).

In contrast to ascomycetes, the transcriptional regulation of lignocellulolytic gene expression in wood-degrading basidiomycetes remains poorly understood, partly due to the absence of clear orthologs of most of the well-studied ascomycete regulators (6). Few basidiomycete TFs have been studied so far, including Ace3/Roc1 and Cre1/CreA orthologs from ascomycetes (14–18). Ace3 in the white-rot fungus *Dichomitus squalens* and Roc1 in the wood-degrading fungus *Schizophyllum commune* regulate genes encoding cellulases, selected xylanases, and cellobiose transporters in response to cellobiose (15, 16). Cre1/CreA acts as a carbon catabolite repressor also in basidiomycetes (14, 18). For lignin degradation, two TFs from the white-rot fungus *Trametes hirsuta*, TH8421 and TH4300, have been linked to the induction of laccase expression in response to specific aromatic monomers (17). Despite these findings, the regulation of hemicellulose-degrading genes in white-rot fungi remains largely unexplored, representing a significant gap in our understanding of fungal plant biomass degradation.

*D. squalens* is a white-rot basidiomycete that naturally colonizes softwoods, in which galactoglucomannans are abundant mannose-containing hemicelluloses (19, 20) . Here, we identify and characterize Bhr1, a previously undiscovered TF, from the white-rot basidiomycete *D. squalens*. Bhr1 exhibits an unusual domain architecture that is predicted to combine a septin-like P-loop NTPase fold with Zn(II)2Cys6 DNA-binding domains and appears to be phylogenetically largely restricted to plant-associated Agaricomycetes. Using CRISPR/Cas9-mediated gene disruption, transcriptomics, and enzyme activity assays, we demonstrate that Bhr1 is a key regulator of (hemi-)cellulase gene expression in *D. squalens*, acting in a substrate-dependent manner with a particularly strong response to mannose-rich hemicelluloses. These results illustrate how diverse regulatory strategies can reflect fungal adaptation to complex plant biomass.

## RESULTS

### Selection of candidate regulators and generation of the CRISPR/Cas9 mutants

Candidate TFs were selected from a previous study focused on glucose-mediated carbon catabolite repression in *D. squalens* (14). In that work, fungal cultures were initially induced using cellulose or xylan and subsequently transferred either to the same substrate or one supplemented with glucose. This resulted in the differential expression of 50 TF-encoding genes in glucose-supplemented media compared to media without glucose (14). From these, we selected the four genes showing the highest fold change (FC) under glucose repression, with their corresponding protein IDs at JGI MycoCosm database being: Dicsqu464_2_2903370 and Dicsqu464_2_3016749 from cellulose cultures, and Dicsqu464_2_2987313 and Dicsqu464_2_3079262 from xylan cultures.

Gene disruption was performed in *D. squalens* wild-type (WT) monokaryotic strain CBS 464.89 (21) using a validated CRISPR/Cas9-editing system for *D. squalens* (22) and custom oligos (Table S1). Nonsense mutations were introduced upstream of the predicted TF domains to prevent expression of functional proteins (Fig. S1). Sanger sequencing confirmed successful disruption of Dicsqu464_2_2903370, Dicsqu464_2_3079262, and Dicsqu464_2_2987313 encoding genes in at least one mutant. Despite multiple attempts, the gene encoding Dicsqu464_2_3016749 could not be edited and was excluded from further analyses.

### Regulatory mutant of *D. squalens* shows a distinct phenotype on hemicellulose

**Fig 1:**
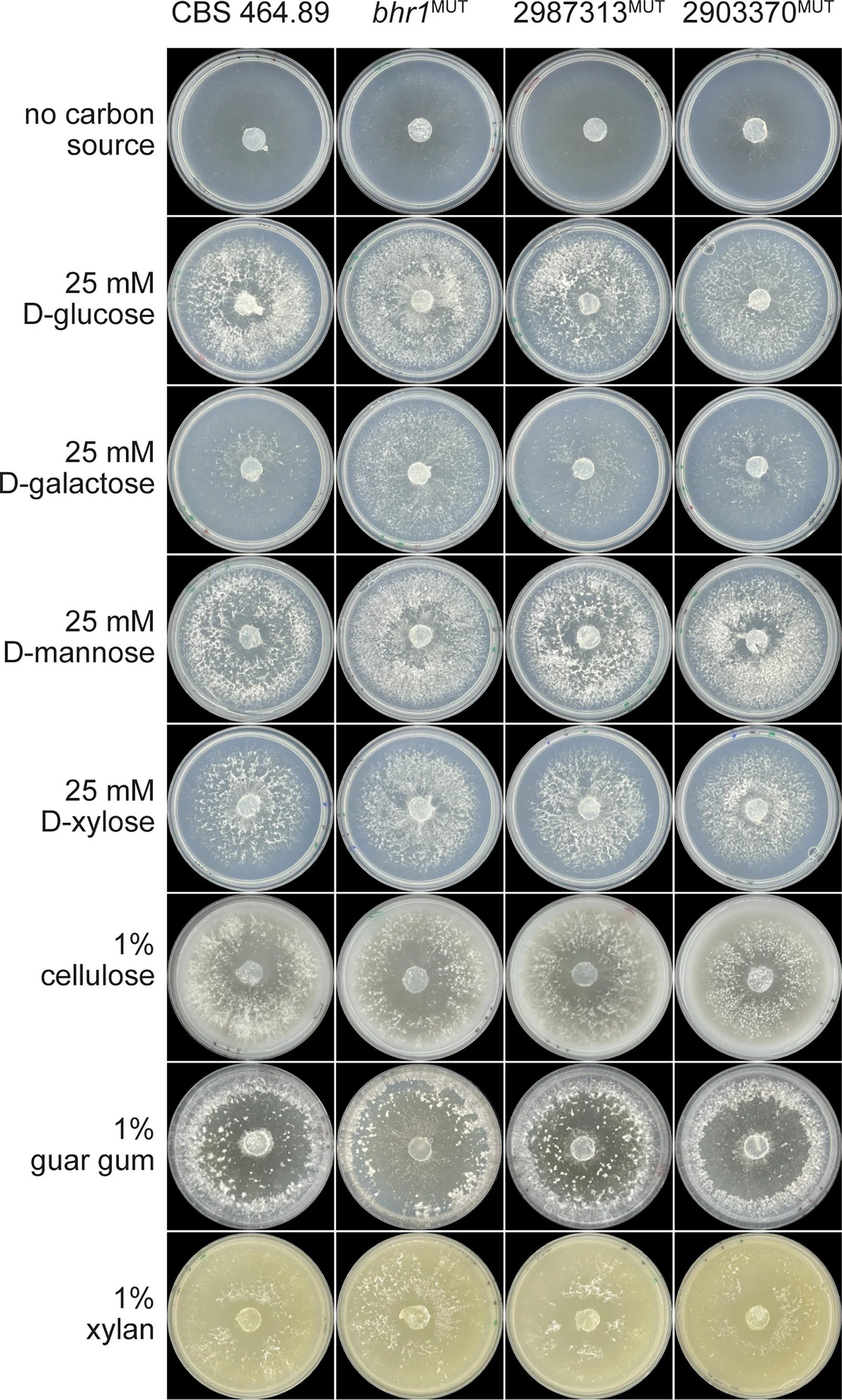
Growth profiling from 5-day cultivations of *Dichomitus squalens* wild-type strain CBS 464.89, *bhr1*^MUT^, 2903370^MUT^, and 2987313^MUT^ on plant biomass-derived mono- and polysaccharides.

To assess the phenotypic impact of TF disruption, WT and mutant strains were grown for 5 days on minimal agar medium supplemented with various mono-, di-, oligo-, and polysaccharides derived from plant biomass (Fig. 1; Fig. S2). Disruption of Dicsqu464_2_3079262 produced a distinct growth phenotype on several hemicellulose-derived substrates, and the corresponding gene was designated basidiomycete hemicellulose regulator 1 (*bhr1*).

The *bhr1* disruption mutant strain (*bhr1*^MUT^) displayed altered colony morphology on D-galactose, D-mannose, and guar gum, including thinner mycelium, lack of aerial hyphal tufts, and irregular surface textures (Fig. 1). Morphology of the *bhr1*^MUT^ strain on other monosaccharides and cellulose- or pectin-derived substrates was similar to the WT, suggesting a substrate-specific response. Mutants of Dicsqu464_2_2903370 (2903370^MUT^) and Dicsqu464_2_2987313 (2987313^MUT^) showed mild or no phenotype differences across all substrates (Fig. 1; Fig. S2) and were not examined further.

### The unique domain architecture of Bhr1 is restricted to plant-associated Agaricomycetes

**Fig 2:**
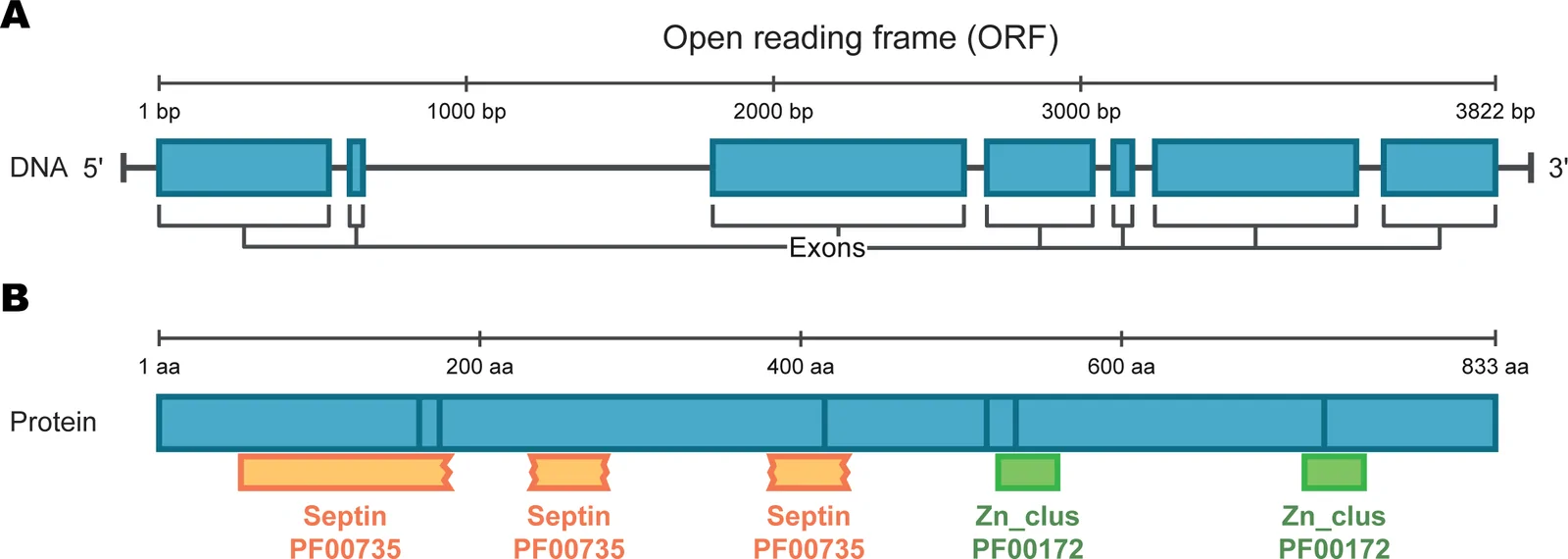
Schematic presentation of (**A**) the gene model and (**B**) the translated peptide of *Dichomitus squalens bhr1* confirmed by Sanger sequencing of cDNA and domain prediction by InterProScan v5.51-85.0, respectively. The orange rectangles depict three partial septin domains (PF00735), and the green rectangles depict two Zn(II)2Cys6 binuclear cluster domains (PF00172).

Although fungal gene model predictions have improved, public annotations can still contain substantial errors (23). To confirm the coding sequence of *D. squalens bhr1*, we designed primers targeting the annotated start and stop codons (Table S1) in the *D. squalens* CBS 464.89 v2.0 genome (15). PCR amplification was performed on cDNA synthesized from pooled RNA collected across multiple time points and conditions in previous studies (24, 25). A single ~2,500 bp amplicon was obtained, and Sanger sequencing verified the annotated gene model, including a coding sequence of 833 amino acids with the predicted exon-intron structure, no evidence of splice variants, and the presence of an unusually large intron of 995 bp (Fig. 2). We acknowledge, however, that the use of the start and stop codon-bound primers cannot exclude alternative start or stop sites outside the annotated boundaries but would require direct mapping of transcript termini by rapid amplification of cDNA ends or long-read transcript sequencing.

Domain analysis of the Bhr1 protein sequence with InterProScan 5 identified three partial septin domains (PF00735) and two Zn(II)2Cys6 binuclear cluster domains (PF00172; Fig. 2). Notably, the Zn(II)2Cys6 finger domains were located closer to the C-terminus, representing a rare configuration for this TF family (7, 26). Despite this unusual placement, conserved cysteines and strong sequence similarity to domain models support their classification as Zn(II)2Cys6 domains.

**Fig 3:**
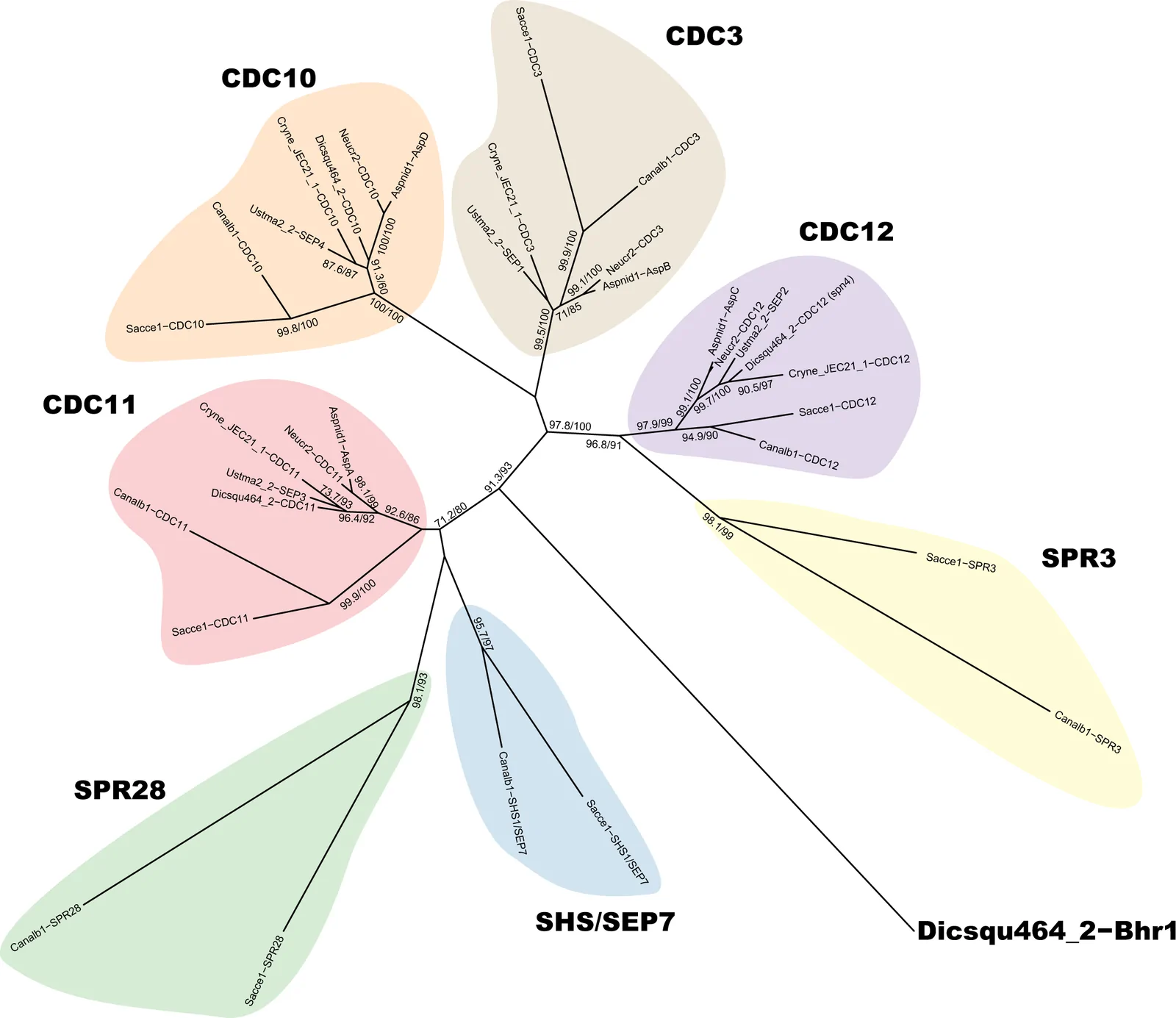
Unrooted maximum-likelihood phylogenetic tree of Bhr1 and the characterized fungal septin gene members of the Bhr1 orthogroup (Table S2). Clades corresponding to *D. squalens* Bhr1 and canonical septin groups CDC3 (brown), CDC10 (orange), CDC11 (red), CDC12 (purple), SPR3 (yellow), SPR28 (green), and SHS/SEP7 (blue) are annotated. JGI genome portal and protein name are shown. Ultrafast bootstrap and SH-like approximate likelihood ratio test support values ≥50 are displayed on branches.

Alignment of Bhr1 with fungal septins from *Saccharomyces cerevisiae*, *Candida albicans*, *N. crassa*, *Cryptococcus neoformans*, and *Ustilago maydis* (CDC3, CDC10, CDC11, CDC12, SHS1, SPR3, and SPR28; Table S2) revealed divergence in the highly conserved septin motifs G1 (GxxGxG[K/R][ST]) and G3 (DT[P/V]GxG), which are essential for GTP binding and hydrolysis (27) (Fig. S3). In Bhr1, the G1 motif lacks one of the three conserved glycines, and the G3 motif lacks all the conserved glycines. Only the G4 motif matched the expected residues. Accordingly, phylogenetic analysis placed Bhr1 on a distinct clade from the fungal septins (Fig. 3). These deviations indicate that the predicted septin domain may represent a divergent septin-like or “pseudo” P-loop NTPase with altered substrate binding (28).

Ortholog analysis across 150 selected fungal genomes (Table S3) performed by OrthoFinder revealed that all proteins in the Bhr1 orthogroup contained at least partial septin domains, while C-terminal Zn(II)2Cys6 domains occurred only in a subset of 41 proteins, all within the Agaricomycetes class of Basidiomycota. Phylogenetic analysis of the orthogroup (Fig. S4) placed *D. squalens* Bhr1 in a basidiomycete-specific clade distinct from canonical fungal septins (Table S2). The species tree showed that the members of this clade were enriched in wood-degrading and mycorrhizal species (Fig. S5), suggesting ecological specialization.

### Disruption of *bhr1* alters (hemi-)cellulase gene expression

To assess transcriptomic effects of *bhr1* disruption, RNA-seq was performed on biological triplicate of *D. squalens* WT and *bhr1*^MUT^ strains grown on minimal agar supplemented with D-glucose, D-mannose, cellulose, guar gum, or xylan for 5 days. Genes with low expression (DESeq2-normalized counts <10 across all samples) were excluded to minimize noise (Table S4A). One WT D-glucose replicate with a Pearson correlation coefficient <0.80 was identified as an outlier and removed from further analysis (Table S5). Differential expression was calculated using WT samples from each substrate as a reference. Across all substrates, 2,200 differentially expressed genes were identified (Table S4B) using false discovery rate (FDR) <0.05 and FC ≥2.5 threshold to focus on strong responders and reduce false positives (24).

Principal component analysis revealed strong separation between WT and mutant samples on D-glucose, D-mannose, and guar gum, but not on cellulose or xylan, indicating substrate-specific transcriptional changes (Fig. S6). In the mutant, xylan-grown samples separated from D-glucose- and D-mannose-grown samples, suggesting that Bhr1-mediated transcriptional regulation is more pronounced on simple sugars and guar gum than on cellulose or xylan.

**Fig 4:**
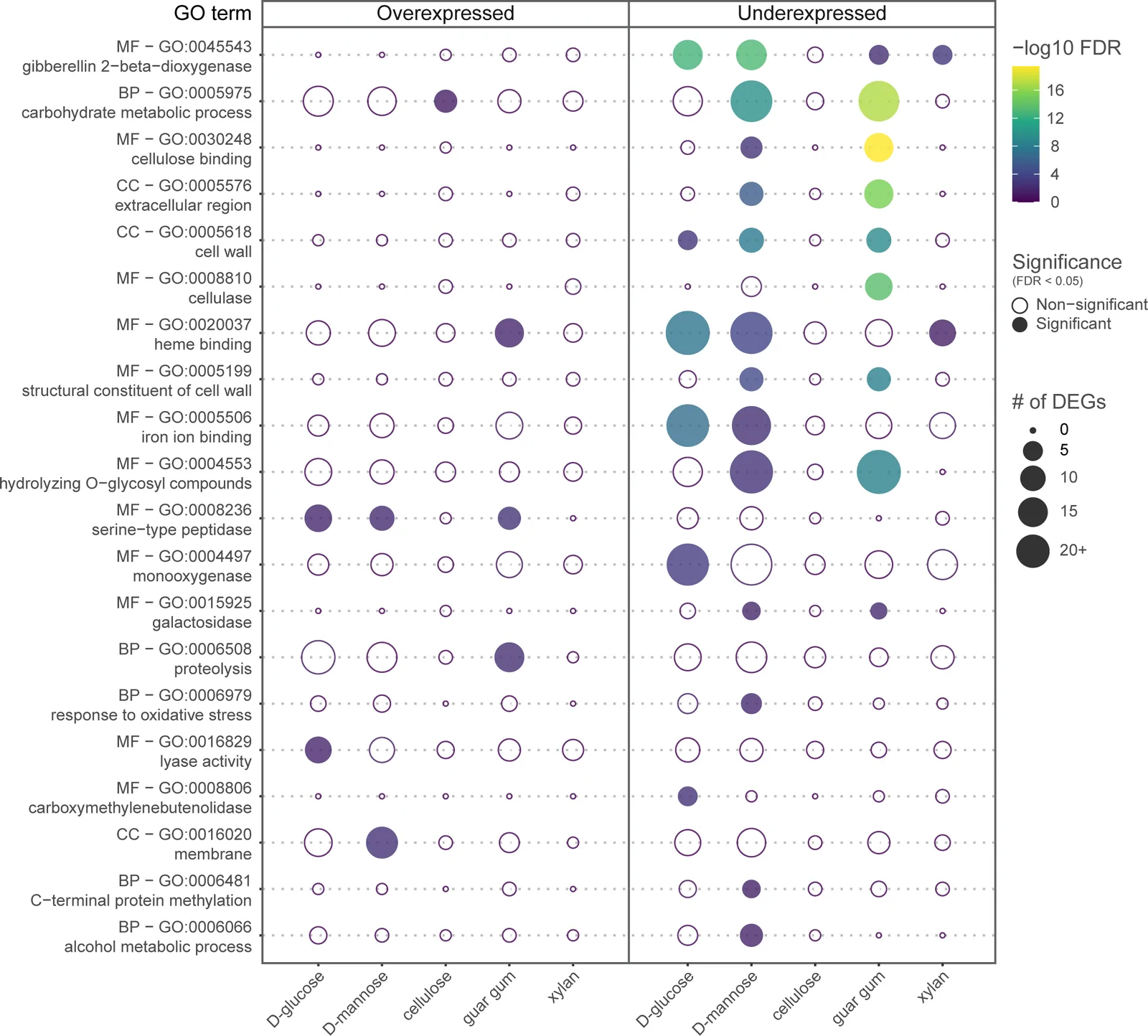
Gene ontology (GO) terms associated with the predicted function of over- and underexpressed genes in *Dichomitus squalens bhr1*^MUT^ compared to the wild-type on plant biomass-derived mono- and polysaccharides. The size and color of the circles represent the gene count and statistical significance of the enriched GO terms, calculated as the false discovery rate (FDR), respectively. Biologically redundant GO terms were manually removed. BP, biological process; CC, cellular component; MF, molecular function; DEG, differentially expressed gene.

Gene ontology (GO) enrichment analysis revealed significant downregulation of carbohydrate metabolism (GO:0005975), cellulose binding (GO:0030248), and cellulase activity (GO:0008810) in *bhr1*^MUT^ on D-mannose and guar gum (Fig. 4). Hydrolase activity on O−glycosyl compounds (GO:0004553) enrichment was similarly reduced, whereas xylan-grown cultures showed no significant enrichment of these terms. Additionally, heme binding (GO:0020037) and iron ion binding (GO:0005506) were also reduced in *bhr1*^MUT^ on D-glucose and D-mannose.

**Fig 5:**
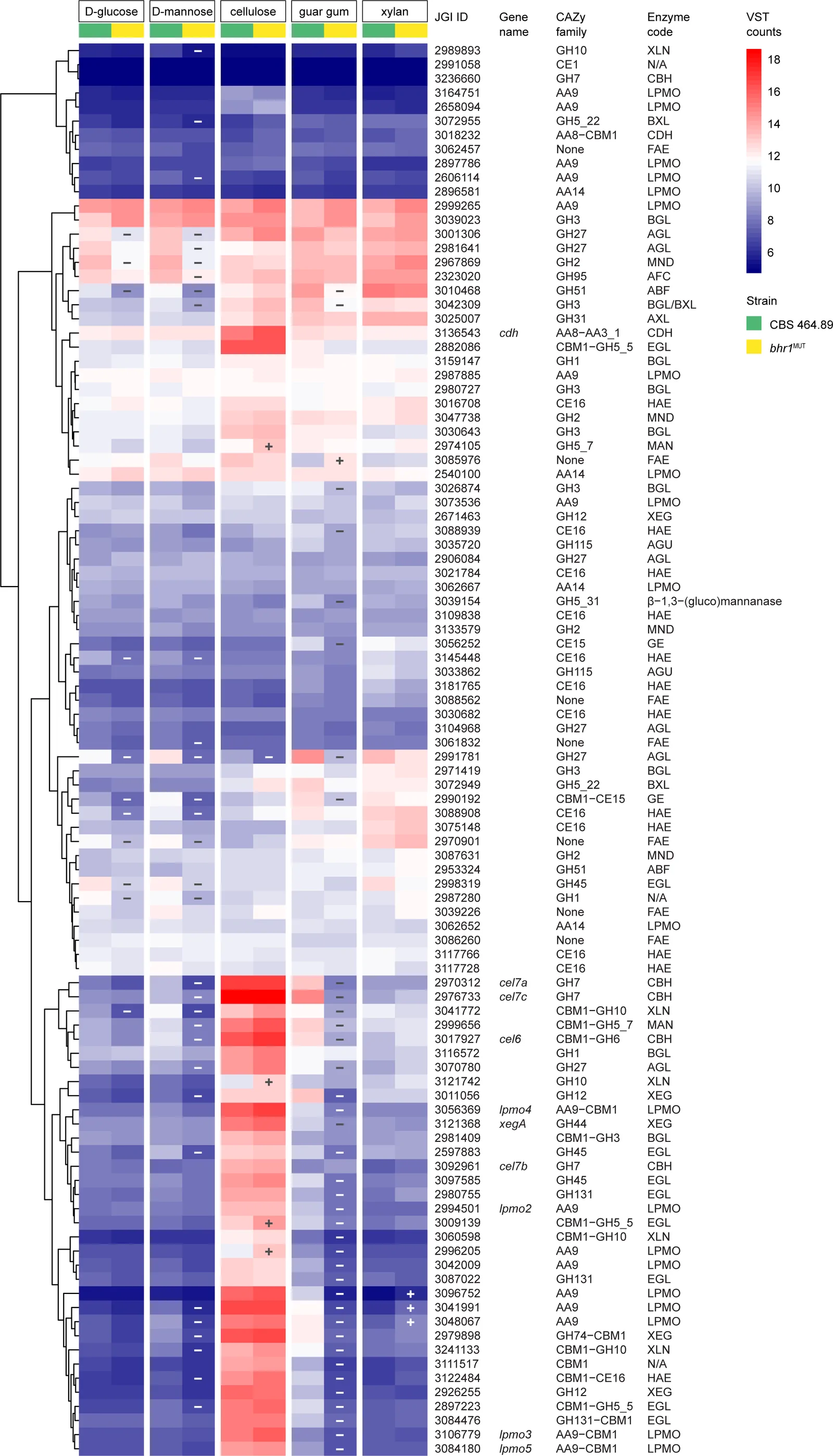
Hierarchical clustering of putative (hemi-)cellulose-active CAZy genes and feruloyl esterases (FAEs) from 5-day cultivations of *Dichomitus squalens* CBS 464.89 and *bhr1*^MUT^. The color scale represents the average variance-stabilized transformed (VST) expression values from biological triplicate. Significantly over- and underexpressed genes compared to the wild type are marked with a plus (+) and minus (−) symbols, respectively. Clustering was performed by complete-linkage and Euclidean distance based on mean VST counts. JGI protein IDs correspond to *D. squalens* CBS 464.89 v2.0 genome annotation available at the JGI MycoCosm database. Enzyme codes are listed in Table S4.

To link the transcriptional changes to biomass degradation, we examined differentially expressed CAZyme-encoding genes (Fig. 5). On guar gum, 38 CAZyme genes predicted to act on cellulose or hemicellulose were underexpressed in the *bhr1*^MUT^, including a β-mannosidase (MAN), a β−1,3-(gluco)mannanase, three endoxylanases (XLNs), four xyloglucan endoglucanases (XEGs), a β-xylosidase (BXL), and nine lytic polysaccharide monooxygenases (LPMOs). This pattern mirrors the growth defects observed on guar gum (Fig. 1). In contrast, xylan-grown cultures showed minimal effects, with only three overexpressed LPMOs and no significant underexpression of cellulases. In the *bhr1*^MUT^ strain, 11 cellulases (β-glucosidases, cellobiohydrolases, and endoglucanases) were underexpressed on guar gum but not on cellulose. These findings indicate that Bhr1 regulates (hemi-)cellulase expression in a substrate-dependent manner, with the strongest effects on mannose-rich hemicelluloses such as guar gum.

Expression analysis of selected *D. squalens* TFs further underscored the distinct role of Bhr1 in (hemi-)cellulase regulation (Fig. S7). In the *bhr1*^MUT^ strain, *bhr1* transcript levels (FPKMs) were significantly higher (*P* <0.05) on the complex substrates cellulose, guar gum, and xylan compared to the WT, whereas its expression in the WT on D-mannose was highly variable. The recently characterized *D. squalens* cellulase activator gene *ace3* (15) showed lower expression in *bhr1*^MUT^ on D-mannose and guar gum but not on cellulose, consistent with the reduced cellulase gene expression observed under these conditions. The carbon catabolite repressor gene *cre1* (14) displayed generally stable expression between strains, except on D-mannose where *bhr1*^MUT^ showed higher FPKMs. In contrast, the remaining candidate TFs showed no clear substrate-dependent patterns. Together, the transcriptional changes observed in the *bhr1*^MUT^ strain include altered expression of *ace3* and *cre1*, whose contribution to the (hemi-)cellulase phenotype, either as direct or downstream targets of Bhr1, remains to be tested.

### Enzyme activities confirm a role for *D. squalens* Bhr1 in (hemi-)cellulase regulation

To functionally validate the role of *D. squalens* Bhr1 in (hemi-)cellulase regulation, extracellular enzyme activities were measured from WT and *bhr1*^MUT^ triplicate cultures grown on cellulose, guar gum, or xylan as the sole carbon source. Crude culture supernatants were collected after 6, 12, 18, and 24 days and used in 3,5-dinitrosalicylic acid (DNS)-based assays to measure cellulase, mannanase, and xylanase activities.

**Fig 6:**
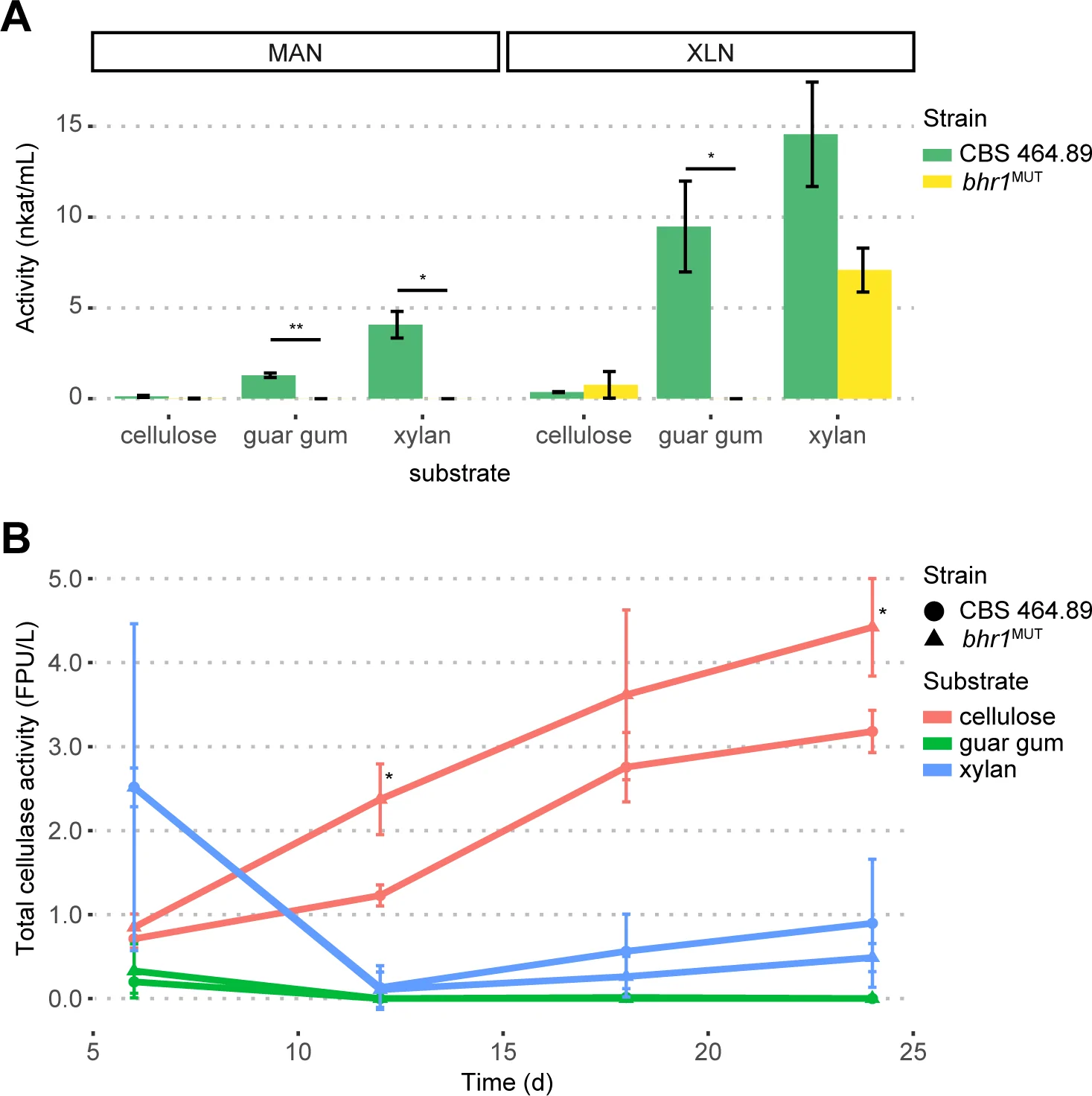
Extracellular (**A**) mannanase (MAN) and xylanase (XLN), and (**B**) total cellulase activity from shaken liquid cultures of *Dichomitus squalens* CBS 464.89 and *bhr1*^MUT^ supplemented with 1% crystalline cellulose, guar gum, or xylan. Error bars represent the standard deviation of biological triplicates. Welch’s *t*-test was used to compare the activities between the strains, and *P* values were labeled as * (≤0.05), ** (≤0.01), and *** (≤0.001). FPU: filter paper units.

Mannanase activity was induced by guar gum and xylan in the WT strain (1.29 ± 0.01 nkat/mL and 4.08 ± 0.74 nkat/mL, respectively) at day 6 but was nearly absent in *bhr1*^MUT^ (Fig. 6A). Similarly, xylanase activity was significantly lower in *bhr1*^MUT^ on guar gum (*P* <0.05; Fig. 6A). In both strains, mannanase and xylanase activities were minimal (<1 nkat/mL) on cellulose, consistent with the substrate specificity of these enzymes. No mannanase or xylanase activity was detected on any substrate after day 6, indicating early and transient induction under the tested conditions.

In contrast to the loss of mannanase and xylanase activity, total cellulase activity was significantly increased in *bhr1*^MUT^ compared to the WT at days 12 and 24 (Fig. 6B). This is consistent with Bhr1 not being required for cellulase induction under cellulose-rich conditions, where the cellulolytic response of *D. squalens* is controlled by *Ds*Ace3 (14, 15). The elevated cellulase activity in *bhr1*^MUT^ may reflect a compensatory response comparable to that observed with the disruption of *Dsace3*, where several sugar metabolic genes were upregulated in the mutant (15). On guar gum and xylan, cellulase activity was generally low in both strains, particularly at later time points. Variability in cellulase activity among triplicate from xylan cultures at day 6 could reflect trace impurities that induce cellulases in the commercial xylan preparation (29) or transient induction via xylan degradation products and regulatory cross-talk.

## DISCUSSION

In this study, we described Bhr1 from the white-rot fungus *D. squalens* as, to the best of our knowledge, the first transcriptional regulator of (hemi-)cellulases in white-rot basidiomycetes, with a particularly prominent role on mannose-rich substrates. *D. squalens* naturally colonizes softwoods, which are rich in mannose-containing hemicelluloses such as galactoglucomannans (30), providing ecological context for the specific regulatory activity of Bhr1. While (hemi-)cellulase regulators have been extensively characterized in ascomycetes, their functional counterparts in basidiomycetes have remained elusive (5, 6). The absence of clear sequence homologs to these ascomycete TFs suggests divergence in the transcriptional control of plant biomass degradation between the two fungal phyla. The distinctive domain architecture of Bhr1, combining a septin-like P-loop NTPase fold with Zn(II)2Cys6 DNA-binding domains, and its restricted distribution within plant-associated Agaricomycetes, may help explain why such regulators have escaped detection in previous studies.

Bhr1 selectively induced genes encoding mannanases, XLNs, BXLs, XEGs, and associated LPMOs on guar gum in *D. squalens*, while leaving their expression on xylan largely unaffected. Since monosaccharides are generally insufficient to induce CAZyme-encoding genes in *D. squalens* (31), a mannose-containing disaccharide or oligosaccharide may act as the physiological signal. The decline in mannanase and xylanase activity after 6 days on guar gum and xylan suggests that (hemi-)cellulase induction is rapid and transient, which could explain why previous transcriptomic studies of *D. squalens* performed after 5 days on solid media (31) failed to capture this response. Similarly, the measurable xylanase activity on xylan despite the lack of differential transcriptional activation could be explained by transient induction of gene expression, differences between solid and liquid culture conditions (32), or post-transcriptional regulation by increasing translation of low-copy transcripts. Together, these findings suggest that Bhr1 plays a sustained role in mannose-rich hemicellulose utilization but a more context-dependent role in xylan degradation.

Consistent with the preferential induction of (hemi-)cellulases on mannose-rich substrates, the WT *D. squalens* showed stronger growth on D-mannose and guar gum than on xylan or D-xylose. In contrast, *D. squalens bhr1*^MUT^ exhibited altered colony morphology on D-mannose and guar gum. By tailoring (hemi-)cellulase induction toward mannose-containing polymers, *D. squalens* Bhr1 may provide a selective advantage in its natural softwood substrates, where galactoglucomannans are abundant components of the hemicellulose matrix (20, 30).

Mechanistically, *D. squalens* Bhr1 exhibits a narrower activation spectrum than the characterized ascomycete Zn(II)2Cys6 regulators (5). In our study, *bhr1* disruption affected (hemi-)cellulase gene expression but left expression of sugar transporter and sugar metabolic genes largely unchanged. This differs from both ascomycete (hemi-)cellulase regulators, e.g., XlnR, AraR, CLR-1/ClrA, and CLR-2/ClrB/ManR (11, 33–37), and basidiomycete cellulase activators, i.e., Roc1/Ace3 (15, 16), which coordinate the induction of CAZyme, sugar transporter, and sugar metabolic genes. Bhr1 also differs in architecture from the recently characterized basidiomycete cellulase and xylanase regulator GlSwi6, an APSES TF from *Ganoderma lucidum* that acts through calcium signaling and MAPK pathways (38). This illustrates that basidiomycetes can regulate the (hemi-)cellulases both by recruiting conserved eukaryotic TF families and by deploying lineage-specific architectural innovations such as Bhr1. Additionally, unlike Ace3, which is suggested to be positively autoregulated in *T. reesei* and *D. squalens* (15, 39), *bhr1* transcripts increased in the disruption mutant, suggesting it is not self-inducing and therefore is subjected to higher-level control or feedback from other regulators. Together, these findings indicate that Bhr1 fine-tunes a subset of CAZymes in response to specific polysaccharide cues, raising the question of how its septin-like P-loop NTPase domain contributes to transcriptional regulation.

The unusual architecture of *D. squalens* Bhr1 points to a lineage-specific regulatory innovation. While septin-like P-loop NTPases are best known for cytoskeletal organization and compartmentalization in cells (40, 41), members of the P-loop NTPase superfamily are also co-opted into regulatory functions (42). In bacteria, MalT and CalR2 each contain an N-terminal P-loop NTPase domain fused to a DNA-binding helix-turn-helix, where ATP binding regulates maltose and calcium-responsive operons, respectively (43, 44). In eukaryotes, the SWI/SNF ATPase Snf2/Swi2 drives ATP-dependent remodeling of promoter accessibility (45). The single-subunit remodeler Mot1 (Snf2/Swi2-related) uses ATP hydrolysis to dissociate TATA‐binding protein from promoter DNA (46), while the TFIIH helicase Ssl2 facilitates promoter opening and start-site scanning (47). Fungal gene-specific regulators can recruit these ATPase machines; for example, FgAreB engages SWI/SNF at target promoters in *Fusarium graminearum*, illustrating a direct interface between lineage-specific TFs and P-loop ATPase remodelers (48). To our knowledge, no fungal protein has been reported to fuse a P-loop NTPase domain with a Zn(II)2Cys6 DNA-binding module. The presence of both modules in Bhr1 therefore represents a novel basidiomycete-specific solution that could directly couple nucleotide-dependent conformational switching to regulate (hemi-)cellulase gene expression.

Our results establish Bhr1 as a regulator of (hemi-)cellulase expression in *D. squalens*, but further studies are needed to elucidate its specific role in the regulatory network of plant biomass-converting enzymes. For example, direct binding of Bhr1 to CAZyme gene promoters to identify its primary regulatory targets is yet to be demonstrated. Additionally, the role of the septin-like P-loop NTPase domain remains unresolved: whether it mediates nucleotide-dependent conformational switching, chromatin association, or nuclear positioning is unknown. While our study focused on *D. squalens*, it will be important to determine whether Bhr1 orthologs in other Agaricomycetes fulfill similar functions or display divergent roles, as has been observed for ascomycete regulators such as XlnR, AraR, and ClrB across fungal lineages (9, 10, 36).

In conclusion, our study identifies Bhr1 as a newly characterized regulator of (hemi-)cellulase expression in basidiomycetes. Its unusual domain architecture and selective activity on mannose-rich substrates in *D. squalens* distinguish it from well-studied ascomycete regulators and from characterized basidiomycete activators, e.g., Roc1 and Ace3. While additional work is required to establish the molecular mechanisms of Bhr1 function and its conservation across species, these findings expand the current understanding of transcriptional control of plant cell wall degradation in white-rot fungi and suggest that basidiomycetes rely on lineage-specific regulators to adapt enzyme production to their ecological niches.

## MATERIALS AND METHODS

### Strains and culture conditions

The *D. squalens* WT strain CBS 464.89 (CBS collection, Westerdijk Fungal Biodiversity Institute, Utrecht, the Netherlands) and disruption mutant strains (this study) were kept on 2% malt extract agar (MEA) at 4°C. Pre-cultures were prepared by transferring 0.5 cm mycelium-covered plugs from MEA to low-nitrogen asparagine-succinate (LN-AS) agar (49) supplemented with 0.05% (wt/vol) glycerol. All experiments were conducted in biological triplicates.

Growth profiling cultures were conducted on LN-AS agar supplemented with 0.05% glycerol; 25 mM of D-glucose (Sigma), L-arabinose (Thermo Scientific), D-fructose (Thermo Scientific), D-galactose (Sigma), D-galacturonic acid (Thermo Scientific), D-mannose (Thermo Scientific), L-rhamnose (Thermo Scientific), D-xylose (Sigma), cellobiose (Sigma), or lactose (Merck); or 1% (wt/vol) polygalacturonic acid (PGAc; Sigma), crystalline cellulose (Supelco), guar gum (Sigma), inulin (Sigma), pectin from apple (Sigma), pectin from citrus (Sigma), starch (Sigma), or beechwood xylan (Carl Roth) as the sole carbon source. Cultures were incubated at 28°C for 3–5 days.

For analysis of total cellulase, mannanase, and xylanase activity, 50 agar plugs from 5-day MEA pre-cultures were homogenized according to Gonzalez Ramos et al. (15). The mycelial suspensions were inoculated in 50 mL of LN-AS broth supplemented with 1% crystalline cellulose (Fluka), guar gum (Sigma), or beechwood xylan (Carl Roth) and incubated in baffled flasks at 28°C and 120 rpm for 24 days.

### CRISPR/Cas9 gene editing

Gene disruptions were designed based on the *D. squalens* CBS 464.89 genome v2.0 available at http://genome.jgi.doe.gov/Dicsqu464_2 (15). Potential CRISPR target sites within the selected gene loci were identified using Geneious Prime v11.1.4, assessing both on-target activity (50) and off-target specificity scores (51). GeneArt Precision gRNA Synthesis Kit (Thermo Scientific) and custom primers (Table S1) were used to synthesize guide RNAs (gRNAs) according to the manufacturer’s instructions. Assembly of ribonucleoprotein (RNP) complexes was performed using Cas9-NLS (Thermo Scientific) as described previously (22). Custom 120 nt single-stranded oligodeoxynucleotides (ssODNs) were designed as homology-directed repair donors and synthesized (Eurofins Genomics, Konstanz, Germany; Table S1).

PEG-mediated co-transformation of RNP complexes and ssODNs targeting *sdi1* and the loci of the selected genes was performed as previously described (22, 52). Transformants were selected on regeneration agar supplemented with 0.5–2 μg/mL carboxin (Sigma) and screened by restriction enzyme assays (Table S1) of gene-specific PCR amplicons using genomic DNA as a template (53). Mutants were verified by Sanger sequencing (Macrogen, Amsterdam, The Netherlands; Fig. S1).

### Gene model confirmation

The pooled total RNA from *D. squalens* CBS 464.89 cultivations on glycerol, vanillin, veratryl alcohol (25), and cellobiose (15) was used as a template for cDNA synthesis. A cDNA library was synthesized using Superscript IV reverse transcriptase (Invitrogen) and custom oligos (Table S1) according to the manufacturer’s instructions. A full-length *D. squalens bhr1* cDNA was amplified using gene-specific custom primers (Table S1) and Q5 High-Fidelity DNA Polymerase (NEB), following the manufacturer’s instructions, and verified by Sanger sequencing.

### Domain and ortholog analysis

Genome-wide ortholog cluster analysis was performed on 150 fungal species, including representatives of the major fungal families within Dikarya and early-diverging fungi (Table S3). Proteomes of the selected species were downloaded from JGI MycoCosm (http://genome.jgi.doe.gov/), and their reported lifestyle was curated from the literature cited on the species portals (Table S3). Ortholog groups were identified using OrthoFinder v2.5.4 (54) as described in Gonzalez Ramos et al. (15). InterProScan v5.51-85.0 (55) was used to annotate protein domains within the Bhr1 orthogroup. Predicted paralog protein sequences and incorrect gene models were removed from the analysis.

Phylogenetic analysis was performed following a previously described pipeline (56). In summary, sequences from the Bhr1 orthogroup were aligned using MAFFT v7.505 (57) and trimmed with TrimAI v1.5.0 (58) to remove poorly conserved regions. Phylogenetic trees were then inferred using IQ-Tree v3.0.1 (59) with the “MFP” model finder option and 1,000 ultrafast bootstrap replicates. *S. cerevisiae* Myo2 (60), a member of the TRAFAC group of P-loop NTPases, was used as an outgroup for the orthogroup phylogenetic tree.

A subset of proteins, including Bhr1 and characterized septins (Table S2), was analyzed following a similar phylogeny inference pipeline as above. In this case, no outgroup was included, and therefore, the phylogenetic tree is presented as unrooted. Specific residue conservation in the highly conserved septin motifs was visualized using ESPript 3.0 (61).

To estimate the coalescent species tree, the 2,109 orthogroups with a single protein member in at least 75% of the 150 species were selected. Phylogenetic trees for each orthogroup were built as described for the Bhr1 orthogroup. Using the phylogenetic trees, a coalescent species tree was estimated with ASTRAL-Pro3 v1.23.3.6 (62) and its default options (Fig. S4).

### RNA isolation, sequencing, and data analysis

For RNA extraction, strains were cultivated on Polycarbonate Track Etched membranes (GVS, Healthcare & Life Sciences) placed on LN-AS agar plates supplemented with 25 mM of D-glucose (Sigma) or D-mannose (Thermo Scientific) or 1% crystalline cellulose (Fluka), guar gum (Sigma), or beechwood xylan (Carl Roth). Fungal mycelia were harvested from 1.5 cm of the colony periphery after 5 days of incubation at 28°C, and total RNA was extracted using a TRIzol-based method (25). RNA quantity and purity were evaluated using a NanoDrop One spectrophotometer (Thermo Scientific), and integrity was determined using an Agilent 2100 Bioanalyzer with the RNA 6000 Nano Kit (Agilent Technologies). Library preparation and sequencing were performed at the Joint Genome Institute (JGI, Walnut Creek, USA) as previously described (63), with minor modifications described in Gonzalez Ramos et al. (15). Two samples did not pass the quality control, i.e., WT on cellulose and *bhr1*^MUT^ on guar gum, and were discarded from the analysis. Reads were mapped to the *D. squalens* CBS 464.89 genome v2.0.

Gene expression and differential expression analysis were performed following a previously established pipeline (24). In summary, differentially expressed genes were identified using DESeq2 v1.30.1 (64, 65) with an FDR <0.05 and FC ≥2.5 and visualized using pheatmap v1.0.12. Hierarchical clustering was performed on the average variance-stabilized transformed expression values from biological triplicate by complete-linkage clustering and Euclidean distance. Predicted annotations for genes encoding CAZymes, TFs, sugar transporters, and sugar metabolic enzymes were retrieved from *D. squalens* CBS 464.89 genome v2.0 and manually curated on prior studies (24, 25, 56, 63, 66).

### Measurement of extracellular enzymatic activities

Total cellulase, mannanase, and xylanase activities from shaken liquid cultures of *D. squalens* CBS 464.89 and *bhr1*^MUT^ grown on cellulose, guar gum, or beechwood xylan were measured in technical triplicate using DNS-based assays in 50 mM Na-citrate buffer (pH 5.0) at 50°C (67). Total cellulase activity was measured using a previously described microplate-based filter paper assay (68). Mannanase and xylanase activities were measured using 0.5% locust bean gum (Fluka) and 1% beechwood xylan (Carl Roth) (69) as substrates and with D-mannose (Thermo Scientific) and D-xylose (Sigma) as standards, respectively. Reactions were incubated for 24 h, 60 min, and 10 min for total cellulase, mannanase, and xylanase assays, respectively, and stopped with 100 µL of DNS reagent. Samples were boiled for 5 min and cooled, and absorbance was measured at 540 nm. Welch’s *t*-test was used to compare the activities between the strains.

## Data Availability

The read counts for each sample of RNA-seq were deposited in NCBI’s Sequence Read Archive under BioProject accession numbers PRJNA1362415, PRJNA1362416, PRJNA1362430, PRJNA1362450, PRJNA1362455, PRJNA1362456, PRJNA1362458, PRJNA1362459, PRJNA1362500, PRJNA1362514, PRJNA1362516, PRJNA1362518, PRJNA1362523, PRJNA1362540, PRJNA1362547, PRJNA1362557, PRJNA1362569, PRJNA1362571, PRJNA1362608, PRJNA1362609, PRJNA1362633, PRJNA1362646, PRJNA1362648, PRJNA1362661, PRJNA1362665, PRJNA1362672, PRJNA1362680, and PRJNA1362698.

## SUPPLEMENTAL MATERIAL

Supplemental figures spectrum.00736-26-s0001.pdf Figures S1 to S7.

Table S1 spectrum.00736-26-s0002.xlsx Custom oligos used in this study.

Table S2 spectrum.00736-26-s0003.xlsx List of characterized septins used in this study.

Table S3 spectrum.00736-26-s0004.xlsx List of proteomes used in this study including taxonomy and lifestyle.

Table S4 spectrum.00736-26-s0005.xlsx Gene expression levels (A) and differential expression (B) on cultivations of *Dichomitus squalens* CBS 464.89 and *bhr1*^MUT^.

Table S5 spectrum.00736-26-s0006.xlsx Pearson correlation coefficient between RNA-seq samples.

ASM does not own the copyrights to Supplemental Material that may be linked to, or accessed through, an article. The authors have granted ASM a non-exclusive, world-wide license to publish the Supplemental Material files. Please contact the corresponding author directly for reuse.
