# Supplementary material for: The novel transcriptional activator Bhr1 combining NTPase and Zn(II)2Cys6 DNA-binding domains controls (hemi-)cellulase response to mannose-rich substrates in the white-rot fungus Dichomitus squalens
Source: Microbiol Spectr. 2026 Jul 24;14(9):e00736-26. doi: 10.1128/spectrum.00736-26 (PMC13532241; doi:10.1128/spectrum.00736-26)
Supplement: Supplementary file 1 [file spectrum.00736-26-s0001.pdf]

The novel transcriptional activator Bhr1 combining NTPase and Zn(II)2Cys6 DNA-binding domains controls (hemi-)cellulase response to mannose-rich substrates in the white-rot fungus *Dichomitus squalens*

Supplementary figures

A

```
                                                       XbaI (+)
bhr1                    ACGTGCCAAGAAGATAACGGTCGCATGCAATTTCTGCAGGTGTAAGTTCAGCCTCGTTCC  2820
ssODN30_bhr1_stop       ------------------------------------------AGTTCAGCCTCGTTCC      16
bhr1_mut1_seq_F.ab1     ACGTGCCAAGAAGATAACGGTCGCATGCAATTTCTGCAGGTGTAAGTTCAGCCTCGTTCC   268
bhr1_mut1_seq_R.ab1     ACGTGCCAAGAAGATAACGGTCGCATGCAATTTCTGCAGGTGTAAGTTCAGCCTCGTTCC   304

bhr1                    CATTCTATCTGGACGCGGGTTCTCACATTCTGCTCGCGCCCGCAGCACGGAAGCTCAAGT  2880
ssODN30_bhr1_stop       CATTCTATCTGGACGCGGGTTCTCACATTCTGCTCGCGCCTCTAGAACGTTAGCTCTAGT    76
bhr1_mut1_seq_F.ab1     CATTCTATCTGGACGCGGGTTCTCACATTCTGCTCGCGCCTCTAGAACGTTAGCTCAAGT   328
bhr1_mut1_seq_R.ab1     CATTCTATCTGGACGCGGGTTCTCACATTCTGCTCGCGCCTCTAGAACGTTAGCTCAAGT   364

bhr1                    GCGATGGCGGCAGACCGGCATGCGGACAGTGCTTCAAACGTTCGAACGCGTGCGACTACA  2940
ssODN30_bhr1_stop       GCGATGGCGGCAGACCGGCATGCGGACAGTGCTTCAAACGTTCG----------------   136
bhr1_mut1_seq_F.ab1     GCGATGGCGGCAGACCGGCATGCGGACAGTGCTTCAAACGTTCGAACGCGTGCGACTACA   388
bhr1_mut1_seq_R.ab1     GCGATGGCGGCAGACCGGCATGCGGACAGTGCTTCAAACGTTCGAACGCGTGCGACTACA   424
```

B

```
                                                           StuI (+)
Dicsqu464_2-2987313     ACGACAAGAAGTTTGCCTGGTGTGTCGAGATCCATGTGTGCTCAGGGAAGCTGCGTTGCT   477
ssODN29_2987313_stop    ------------------------------------------CAGGGAAGCTGCGTTGCT    18
2987313_mut1_seq_F.ab1  ACGACAAGAAGTTTGCCTGGTGTGTCGAGATCCATGTGTGCTCAGGGAAGCTGCGTTGCT   446
2987313_mut1_seq_R.ab1  ACGACAAGAAGTTTGCCTGGTGTGTCGAGATCCATGTGTGCTCAGGGAAGCTGCGTTGCT   479

Dicsqu464_2-2987313     CAATCTAGCCCGAAGTGAGTCCTGCATTAAGGGGCACCGCTCGTCCGGCTGTCAGCATGC   537
ssODN29_2987313_stop    CAATCTAGCCCGAAGTGAGTCCTGCATTAAGGGGCATCGCTAGGCCTGCTGACAGCATGC    78
2987313_mut1_seq_F.ab1  CAATCTAGCCCGAAGTGAGTCCTGCATTAAGGGGCATCGCTAGGCCTGCTGACAGCATGC   506
2987313_mut1_seq_R.ab1  CAATCTAGCCCGAAGTGAGTCCTGCATTAAGGGGCATCGCTAGGCCTGCTGACAGCATGC   539

Dicsqu464_2-2987313     GGATCGGCCGCTCTTCGAGGTGAAGAAGAAGGGCAGACCGGTGTCCCAATGTGACAAGTG   597
ssODN29_2987313_stop    GGATCGGCCGCTCTTCGAGGTGAAGAAGAAGGGCAGACCGGT------------------   138
2987313_mut1_seq_F.ab1  GGATCGGCCGCTCTTCGAGGTGAAGAAGAAGGGCAGACCGGTGTCCCAATGTGACAAGTG   566
2987313_mut1_seq_R.ab1  GGATCGGCCGCTCTTCGAGGTGAAGAAGAAGGGCAGACCGGTGTCCCAATGTGACAAGTG   599
```

C

```
Dicsqu464_2-2903370     ACCCTCGCGCGCGGCCCCGGCTCTTACCGCAGCGCCCCACCCATGCCGCCCGCAGAGCGG   540
ssODN32_2903370_stop    -------------------GCTCTTACCGCAGCGCCCCACCCATGCCGCCCGCAGAGCGG    41
2903370_mut1_seq_F.ab1  ACCCTCGCGCGCGGCCCCGGCTCTTACCGCAGCGCCCCACCCATGCCGCCCGCAGAGCGG   480
2903370_mut1_seq_R.ab1  ACCCTCGCGCGCGGCCCCGGCTCTTACCGCAGCGCCCCACCCATGCCGCCCGCAGAGCGG   520
                              HindIII (-)
Dicsqu464_2-2903370     CGGGGAAGCTTTGGCCTCGCGACGGCAGGTCAGTTCATCGACGGCGCGCGCGTCGACATG   600
ssODN32_2903370_stop    CGGGGAAGCTAGGGCCTCGCGACGTGAGGTCAGTTCATCGACGGCGCGCGCGTCGACATG   101
2903370_mut1_seq_F.ab1  CGGGGAAGCTAGGGCCTCGCGACGTGAGGTCAGTTCATCGACGGCGCGCGCGTCGACATG   540
2903370_mut1_seq_R.ab1  CGGGGAAGCTAGGGCCTCGCGACGTGAGGTCAGTTCATCGACGGCGCGCGCGTCGACATG   580

Dicsqu464_2-2903370     TATGGCACCGGGACGCCCGCACGGTACGTCAACGCAGGCGCCTCGGCATCGTACCCGCCG   660
ssODN32_2903370_stop    TATGGCACCGGGACGCCCG-----------------------------------------   161
2903370_mut1_seq_F.ab1  TATGGCACCGGGACGCCCGCACGGTACGTCAACGCAGGCGCCTCGGCATCGTACCCGCCG   600
2903370_mut1_seq_R.ab1  TATGGCACCGGGACGCCCGCACGGTACGTCAACGCAGGCGCCTCGGCATCGTACCCGCCG   640
```

Figure S1. Sanger sequencing results of the target loci for CRISPR/Cas9 gene editing of putative transcription factor-encoding genes A) *bhr1*, B) Dicsqu464_2_2987313, and C) Dicsqu464_2_2903370. Point mutations are highlighted in yellow. Modified restriction sites for screening are underlined and shown in blue. Nonsense mutations are underlined in red.

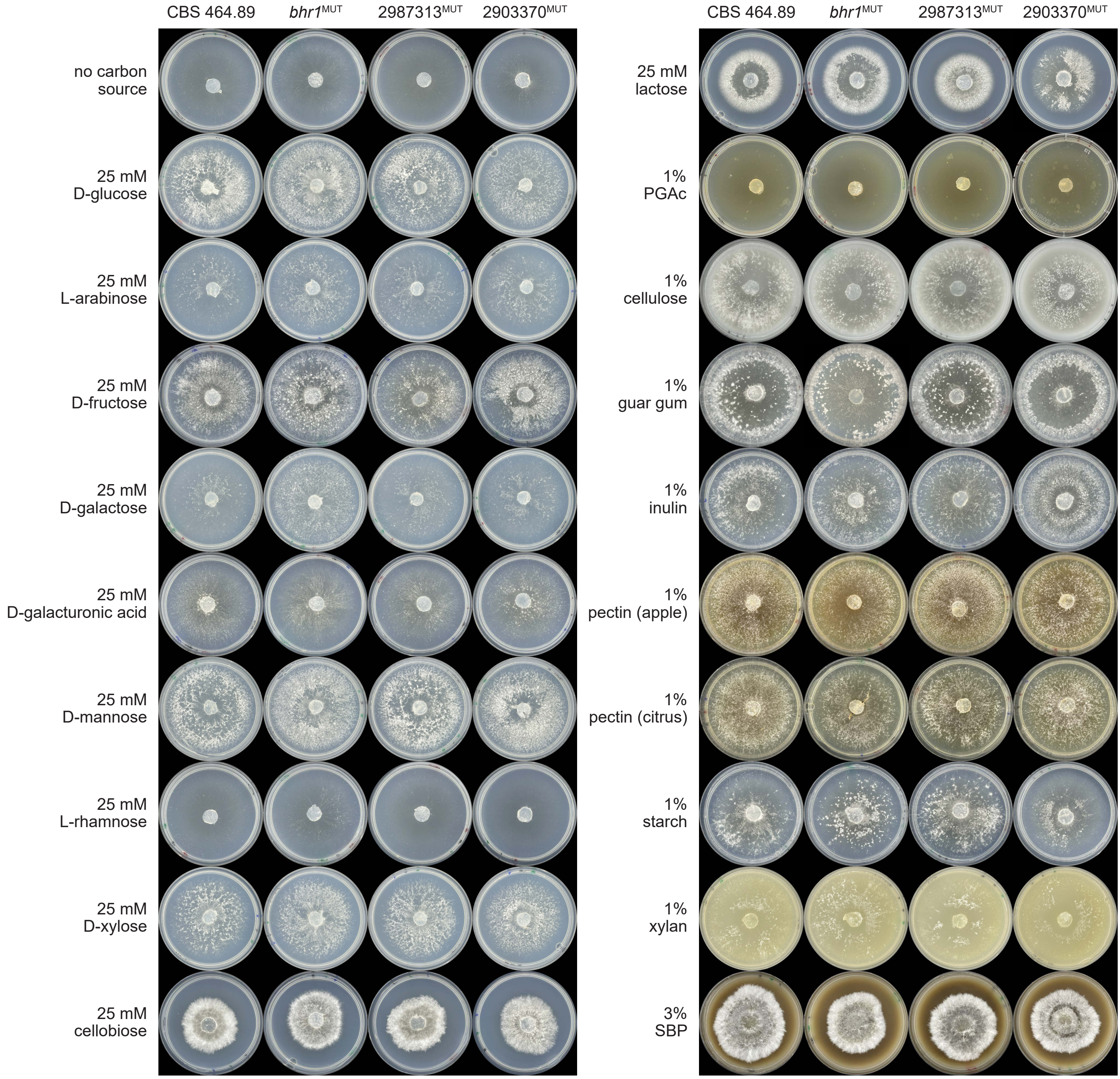

Figure S2. Extended growth profiling from 5-day cultivations of the *Dichomitus squalens* wild-type strain CBS 464.89, *bhr1*^{MUT}, 2903370^{MUT} and 2987313^{MUT} on plant biomass-derived mono-, di-, and polysaccharides. PGAc: polygalacturonic acid; SBP: sugar beet pulp.

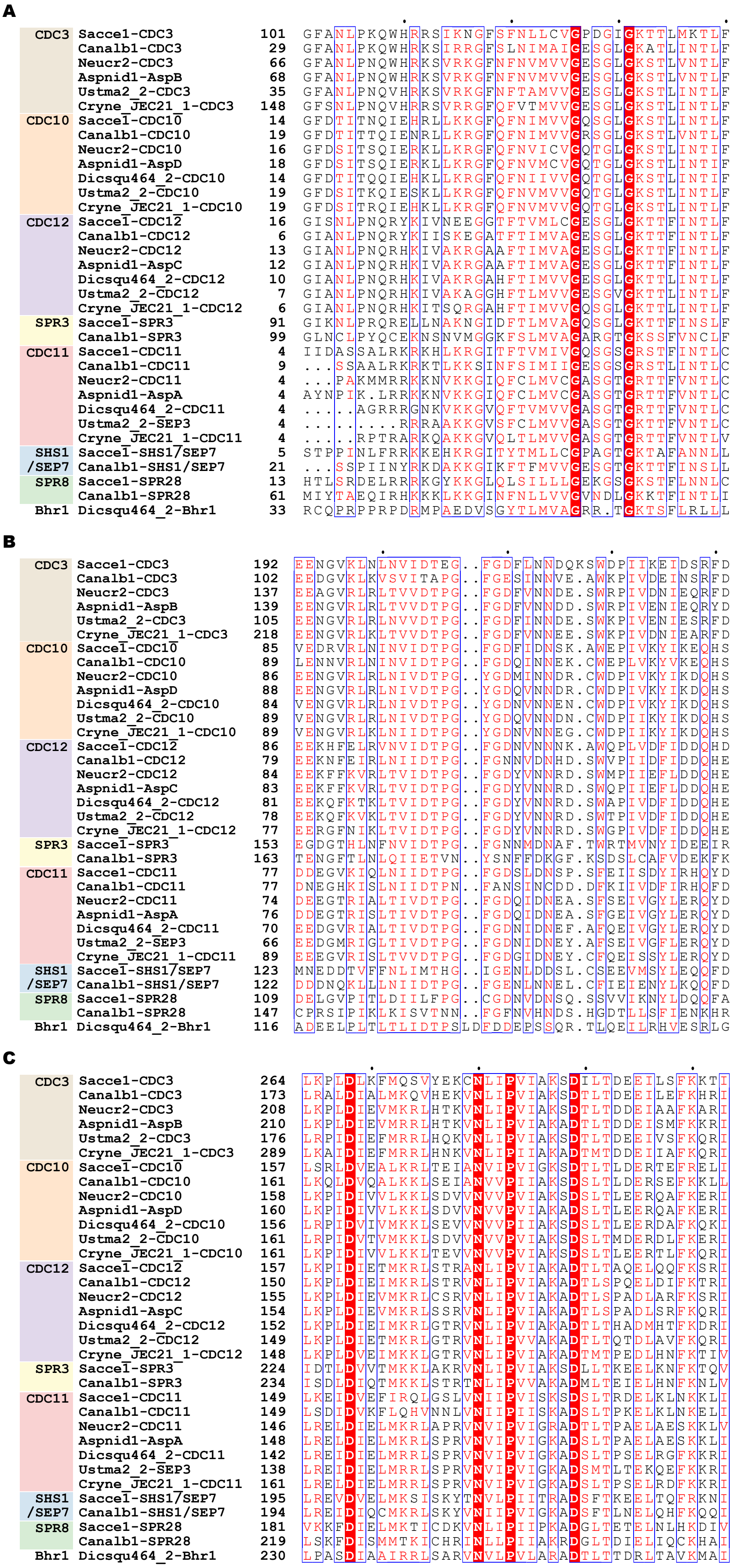

Figure S3. Sequence alignment of the highly conserved septin motifs A) G1, B) G3, and C) G4 of *Dichomitus squalens* Bhr1 and characterized fungal septins (Table S3). Groups corresponding to *D. squalens* Bhr1 and canonical septin groups CDC3 (brown), CDC10 (orange), CDC11 (red), CDC12 (purple), SPR3 (yellow), SPR28 (green), and SHS/SEP7 (blue) are annotated. JGI genome portal and protein name are shown. Alignments were visualized with ESPript 3.0.

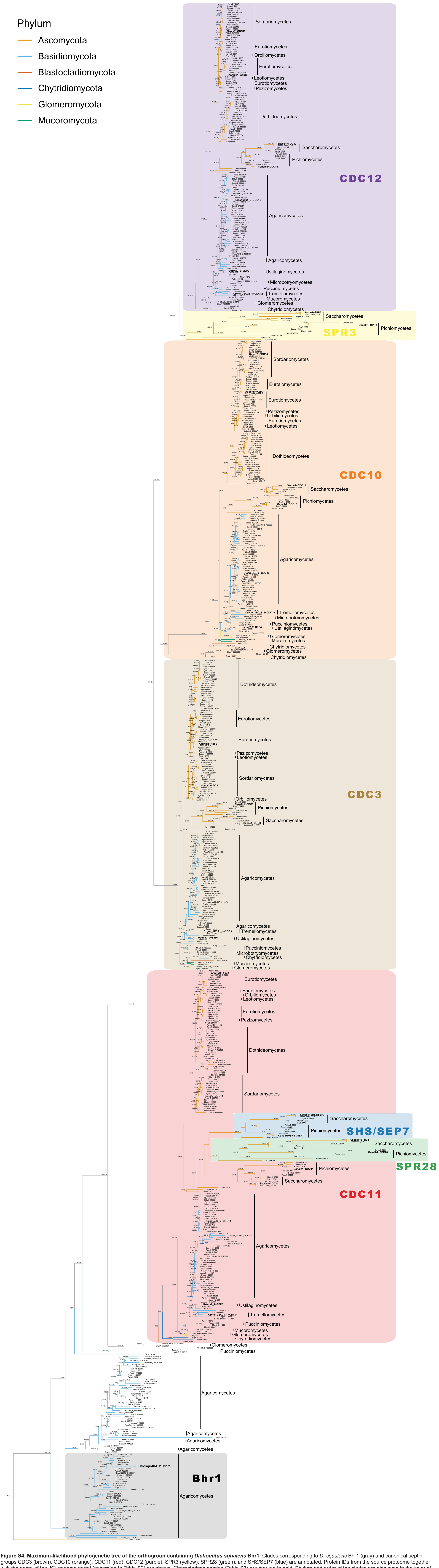

Figure S4. Maximum-likelihood phylogenetic tree of the orthogroup containing *Dichomitus squalens* Bhr1. Clades corresponding to *D. squalens* Bhr1 (gray) and canonical septin groups CDC3 (brown), CDC10 (orange), CDC11 (red), CDC12 (purple), SPR3 (yellow), SPR28 (green), and SHS/SEP7 (blue) are annotated. Protein IDs from the source proteome together with the name of the JGI genome portal (according to Table S2) are shown. Characterized septins (Table S3) are marked in bold. Phylum and order of the clades are displayed in the color of the branches and the labels to the right of the tree, respectively. Ultrafast bootstrap and SH-like approximate likelihood ratio test support values ≥50 are displayed on branches.

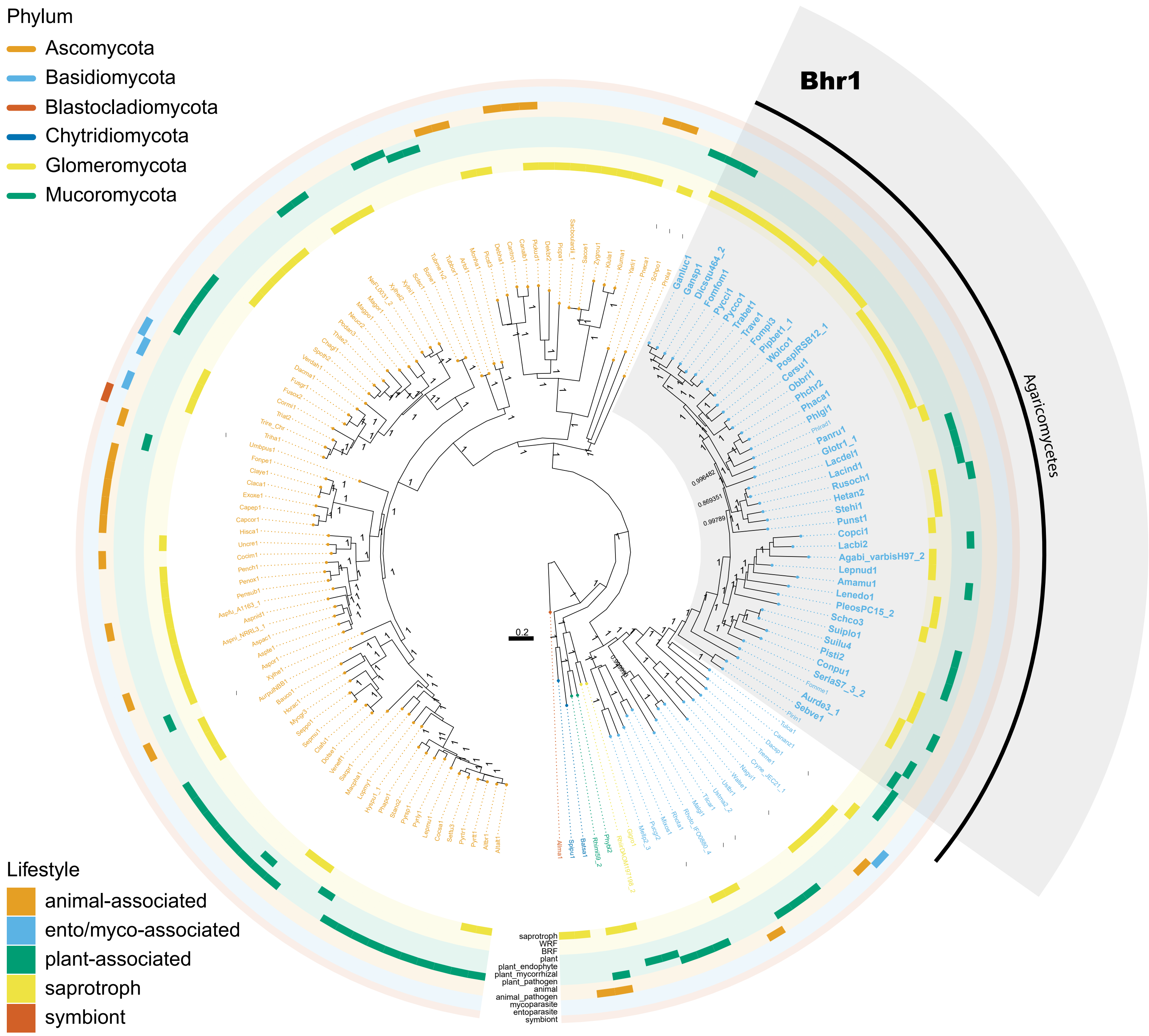

Figure S5. Phylogenomic tree of 150 fungal proteomes with their reported lifestyles. From the genome-wide orthology analysis, 2,109 orthogroups with a single protein member in at least 75% of the proteomes were selected. The corresponding orthogroup phylogenies (IQ-Tree v3.0.1) inferred from the trimmed alignments (MAFFT v7.505; Trimal v1.5.0) were used to estimate the coalescent species tree with ASTRAL-Pro3 v1.23.3.6. Reported lifestyle and name of the JGI genome portal (according to Table S2) are shown. Species containing a member of the Bhr1 clade are marked in bold. The Agaricomycetes clade to which Bhr1 is restricted is highlighted in gray. Phylum-level affiliation of the clades is indicated by text and branch color. Local posterior probabilities ≥0.50 are displayed on branches.

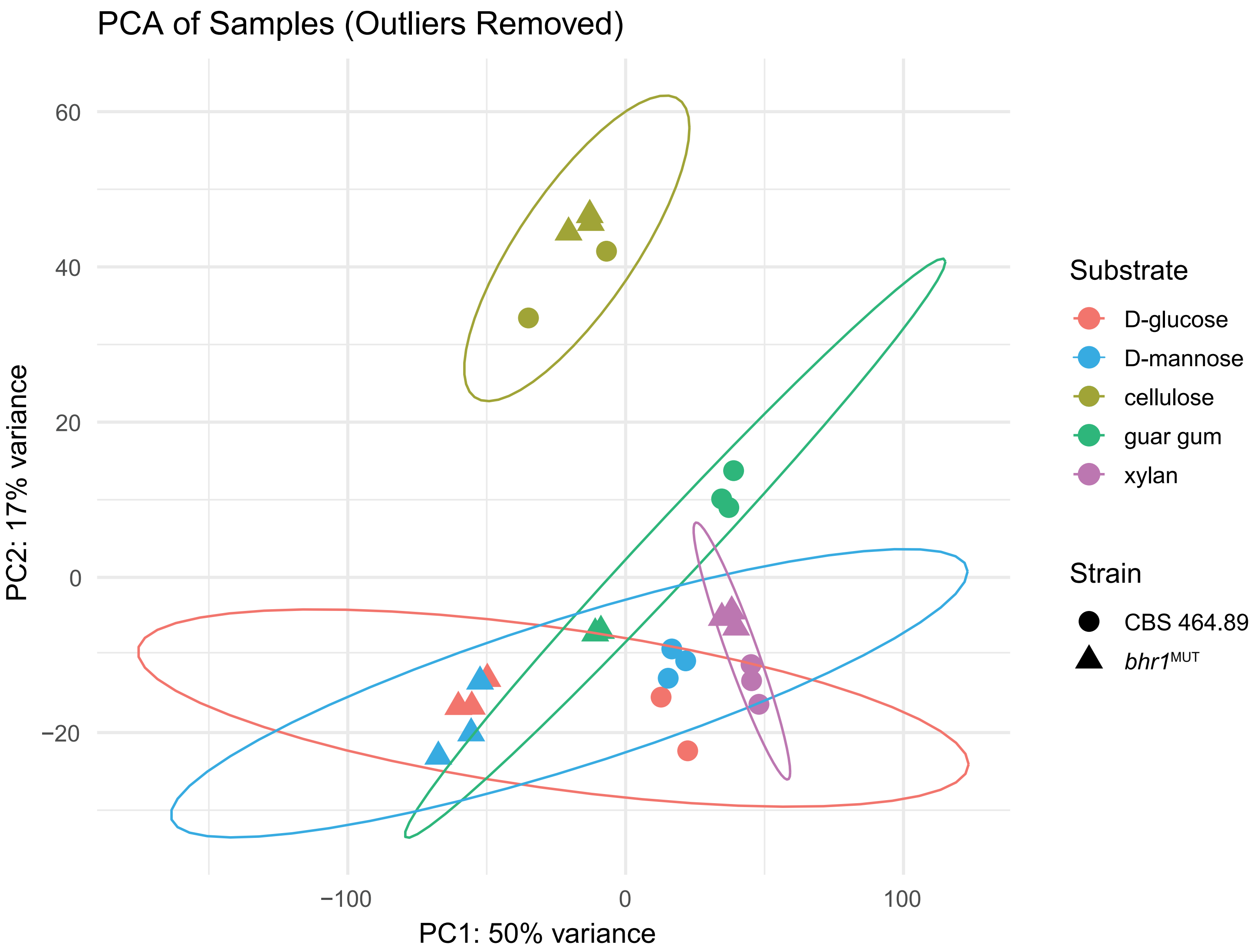

Figure S6. Principal component analysis of the variance stabilized transformed expression values from transcriptomes of *Dichomitus squalens* CBS 464.89 and *bhr1*^{MUT} on plant biomass-derived mono- and polysaccharides, grouped by substrate. Ellipses correspond to 0.05 confidence intervals.

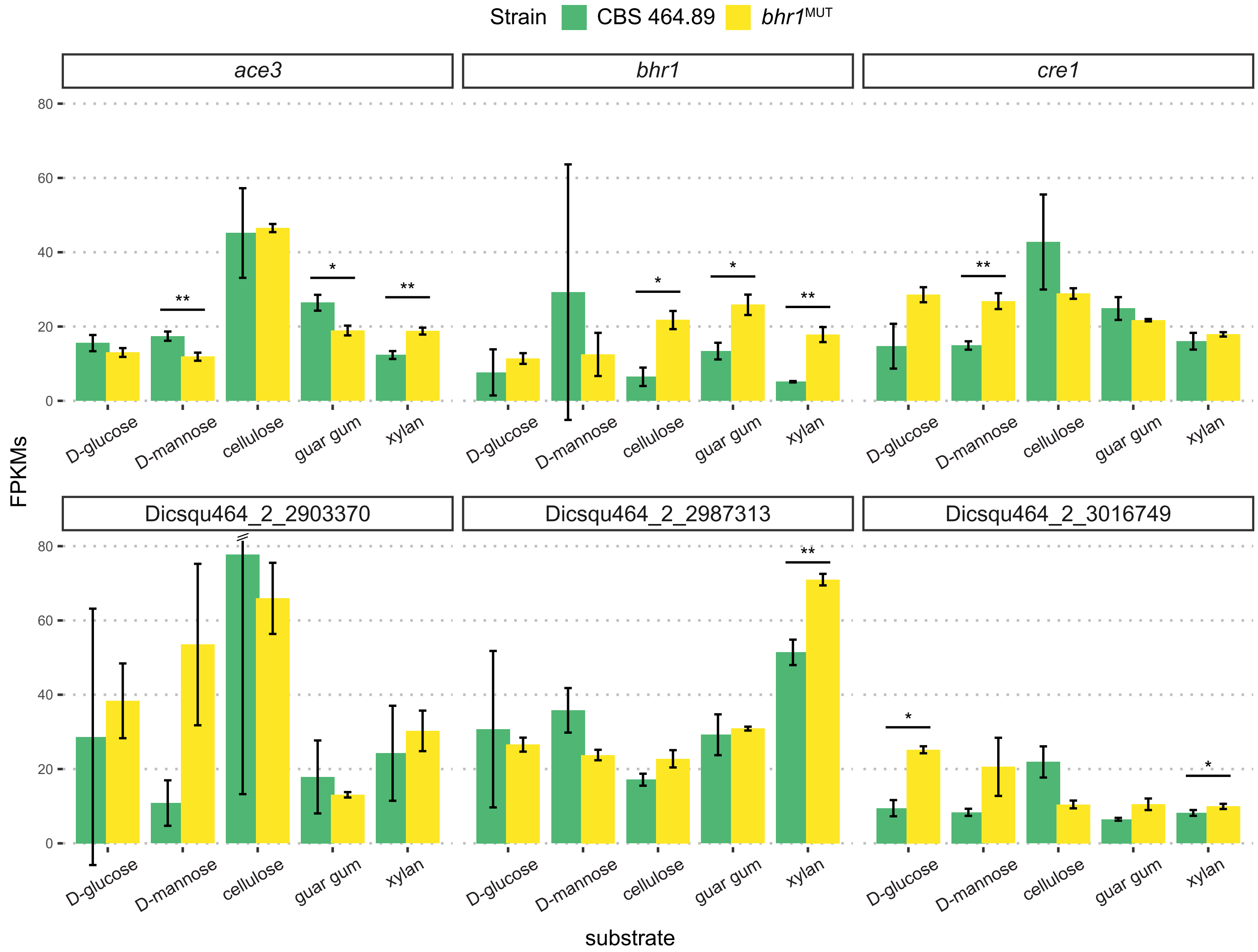

Figure S7. Gene expression levels of selected transcription factor-encoding genes on 5-day cultivations of *Dichomitus squalens* CBS 464.89 and *bhr1*^{MUT}. Values are mean FPKMs from biological triplicates and error bars show the standard error of the mean. Welch's t-test was used to compare the expression of genes between the strains and p-values were labeled as * (≤0.05), ** (≤0.01), and *** (≤0.001).
